# *Dirofilaria immitis* in wolves recolonizing northern Italy: are wolves competent hosts?

**DOI:** 10.1186/s13071-020-04353-2

**Published:** 2020-09-22

**Authors:** Barbara Moroni, Luca Rossi, Pier Giuseppe Meneguz, Riccardo Orusa, Simona Zoppi, Serena Robetto, Francesca Marucco, Paolo Tizzani

**Affiliations:** 1 Dipartimento di Scienze Veterinarie, Università degli Studi di Torino, Largo Braccini 2, 10095 Grugliasco, Italy; 2 Istituto Zooprofilattico Sperimentale Piemonte, Liguria e Valle d’Aosta, Centro di Referenza Nazionale Malattie Animali Selvatici (CeRMAS), S.C. Valle d’Aosta- S.S. Patologie della Fauna Selvatica, Rue de l’Amerique 7G, 11020 Quart, AO Italy; 3 Istituto Zooprofilattico Sperimentale Piemonte, Liguria e Valle d’Aosta via Bologna 148, 10154 Torino, Italy; 4 Centro di Referenza regionale Grandi Carnivori, Ente di Gestione Aree Protette delle Alpi Marittime, Piazza Regina Elena 30, 12010 Valdieri, CN Italy

**Keywords:** *Dirofilaria immitis*, Wolf, Heartworm, Wildlife, Dirofilariasis, Embryogram

## Abstract

**Background:**

Wild carnivores such as the grey wolf (*Canis lupus*), red fox (*Vulpes vulpes*) and golden jackal (*Canis aureus*) are recognized hosts of *Dirofilaria immitis*. However, few studies have focused on their actual role in the epidemiology of heartworm infection. This study describes the prevalence and distribution of *D. immitis* in wolves in a heartworm-endemic area in northern Italy where wolves have recently returned after long-time eradication, and investigates the fertility status of the collected adult nematodes.

**Methods:**

In the frame of a long-term wolf monitoring programme in northwestern Italy, 210 wolf carcasses from four provinces were inspected for the presence of filarioid nematodes in the right heart and pulmonary arteries. Female heartworms were measured, and their uterine content analyzed according to a previously described “embryogram” technique.

**Results:**

Three wolves, all originating from a single province (Alessandria), were positive for *D. immitis* (1.42%, 95% CI: 0.48–4.11%, in the whole study area; 13.6%, 95% CI: 4.7–33.3%, limited to the single province from which infected wolves originated). Mean intensity was 5 worms (range: 3–7) and the female worms measured 21–28 cm in length. Six out of 9 female worms harbored uterine microfilariae: 5 were classified as gravid; 1 showed a “discontinuous gradient”; and 3 were non-gravid.

**Conclusions:**

The present data show that heartworm infection is already prevalent in wolves that have recolonized the known heartworm-endemic area. Based on “embryogram” results, wolves were shown suitable heartworm hosts. Interestingly, investigated wolves appeared similarly exposed to heartworm infection as sympatric unprotected dogs (owned dogs that have never received any heartworm prevention treatment) sampled at the beginning of the wolf return process.
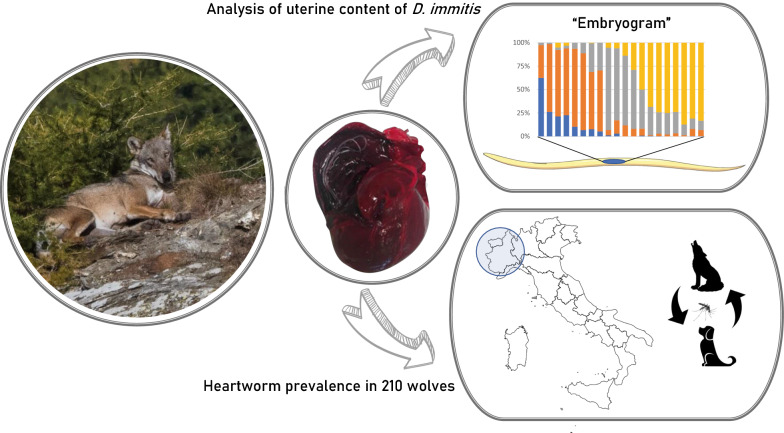

## Background

Heartworm disease is a cardio-pulmonary pathology affecting dogs and other mammalian carnivores worldwide. It is caused by *Dirofilaria immitis*, a large-sized nematode transmitted by several mosquito vectors. There is broad consensus that feral and owned untreated dogs are the main reservoirs for this parasite [1]. Heartworm infection is endemic in dogs in northwestern Italy with the highest prevalence in the humid lowlands (e.g. the Po River Valley) and nearby hills [2–4]. However, in the surrounding mountain areas (western Alps and northern Apennines), only 0.2% and 7.8% of unprotected dogs (owned dogs that have never received any heartworm prevention treatment) tested heartworm-positive, respectively [2]. In northern Italy, heartworm is also commonly found in the abundant red fox (*Vulpes vulpes*), but its contribution to the spread of *D. immitis* is deemed minor, due to the low mean abundance, the high rate of immature worms and the rare gravid females found [5, 6].

The role of wildlife in the maintenance and spread of *D. immitis* is a matter of growing speculations; it is not clear whether wildlife may act as a reservoir [1, 7–9], a sentinel [10] or an accidental host, and which carnivore species are major players in the sylvatic cycle, if any. Several carnivores have been described as heartworm definitive hosts in wildlife: coyote (*Canis latrans*); red fox (*V. vulpes*); grey fox (*Urocyon cinereoargenteus*) [5, 11, 12]; golden jackal (*Canis aureus*) [7, 9, 13]; red wolf (*Canis rufus*) [14]; European wildcat (*Felis silvestris*) [13]; and Eurasian otter (*Lutra lutra*) [13, 15]. Data on heartworm infection in the grey wolf (*Canis lupus*), the closest wild relative of the domestic dog, are still scarce, having been reported only sporadically in Europe since 2001 [7, 16–23].

Wolves were extirpated in northern Italy in the late 1920s [24]; however, individuals dispersing from the population survived in peninsular Italy reappeared in the area since the early-1980s [25]. Since then, these wolves have rapidly grown to a minimum of 300 individuals by winter 2017/2018 [26, 27]. Centralization of wolf necropsies at the Veterinary School in Turin offered the opportunity to investigate, in a large wolf sample, the distribution and prevalence of several infectious and parasitic agents including heartworm, and allowed comparison with data from sympatric domestic dogs. Specifically, in this study we aimed: (i) to provide baseline data on the prevalence and distribution of *D. immitis* in wolves in recently recolonized northwestern Italy; (ii) to investigate, for the first time to our knowledge, the fertility of adult heartworms obtained from naturally infected wolves; and (iii) to evaluate the hypothesis that wild canids in northwestern Italy show an infection prevalence consistent with the prevalence reported in dogs in the endemic areas [2], and that wolves are competent hosts, hence potential reservoirs, of this parasite.

## Methods

During the period 2001–2019, 210 wolves originating from northwest Italy (provinces of Cuneo, Torino, Aosta and Alessandria) were necropsied at the Department of Veterinary Science, University of Turin. Carcasses were classified into three age classes based on body mass and tooth wear [28]: juvenile (< 1 year-old); subadult (1–2 years-old); and adults (≥ 2 years-old). Sex was also recorded as well as the altitude of the sampling location. In particular, the wolves were sampled along an altitude gradient ranging from 100 to around 2500 meters above sea level. “Mountain areas” are defined in the present work as zones with altitude above 600 meters above sea level. The body condition was classified into three categories based on subcutaneous and visceral fat deposits: optimal status; moderate status; and malnutrition. After the removal of lungs and heart from the thoracic cavity, the right chambers of the heart were opened, and pulmonary arteries were carefully inspected for the presence of filarioid nematodes. Helminths were counted and stored in 70% ethanol. Blood microfilaremia in heartworm positive wolves was not investigated due to the poor preservation of the carcasses. Helminths were identified by morphological and morphometric features [29], measured in length and separated by sex. Females were then rehydrated in sterile saline solution at 4 °C for 5 days, and submitted to quantitative analysis of the uterine contents (“embryogram”), according to Lok et al. [30] with minor adaptations. Embryonic stages were attributed to four categories: “pre-larva”; “developed embryo”; “pretzel”; and “stretched microfilaria”. They were counted and then expressed as percentage in each category in every of the 20 equal segments, into which the body of the nematode was divided, from the head (segment 1) to the tail end (segment 20). The segment 1 corresponds to the genital pore. Based on the count of the embryonic stages, five qualitative attributes of reproductive status were assigned to each female specimen, as described by Lok et al. [31]: (i) “normal”, worms with a progressive gradient of embryonic stages from pre-larva in segment 20 to pretzels and microfilariae in segment 1; (ii) “low production of immature forms”, worms with a low number of pretzels and microfilariae; (iii) “discontinuous gradient”, one or more embryonic stages absent; (iv) “microfilariae retained”, females with high numbers of microfilariae in antero-central segments; and (v) “non-gravid”, worms showing only pre-larvae.

Data in this study were compared with previously published data obtained from a large sample of unprotected dogs originating from the same region and provinces. In the study by Rossi et al. [2], the sampling location was attributed to one out of five ecogeographic ranges, as follows: plains; hills; pre-Alps; Alps; and Apennines. The last three were defined as mountain areas, characterized by lower temperatures, higher rainfall and forest canopy, and lower human population density. Extremes for all parameters are found in the Alps.

The descriptive analysis, including 95% confidence intervals (95% CI) using Wilson score for the prevalence estimates, was conducted using the open source software OpenEpi [32]. Other statistical analysis and graphical representation have been carried out with R 3.5.0 [33]. *P*-values < 0.05 were considered statistically significant. Spatial analysis was performed using QGIS software 3.2.0 “Bonn” [34].

## Results

Table 1 shows the distribution of the 210 sampled wolves by sex, age and infection status. A total of 15 specimens of *D. immitis* (9 females and 6 males) were collected from 3 female wolves in January 2016, April 2017 and March 2019 (prevalence: 1.4%; 95% CI: 0.5–4.1%; mean intensity: 5; range: 3–7). One of the infected wolves was a subadult and two were adults. Overall, adults were more infected (marginal significance) than younger wolves (Mid-P exact test, *P* = 0.07, OR: 6.8).

**Table 1:** Age, sex distribution and positivity to heartworm infection of 210 wolves recovered between 2001–2019 in northwest Italy

Age class	Female	Male	Total	Heartworm-positive
	*n* (%)	*n* (%)	*n*	*n*
Juvenile	35 (43.7)	45 (56.2)	80	0
Subadult	42 (51.8)	39 (48.1)	81	1
Adult	23 (46.9)	26 (53.0)	49	2

**Fig. 1:**
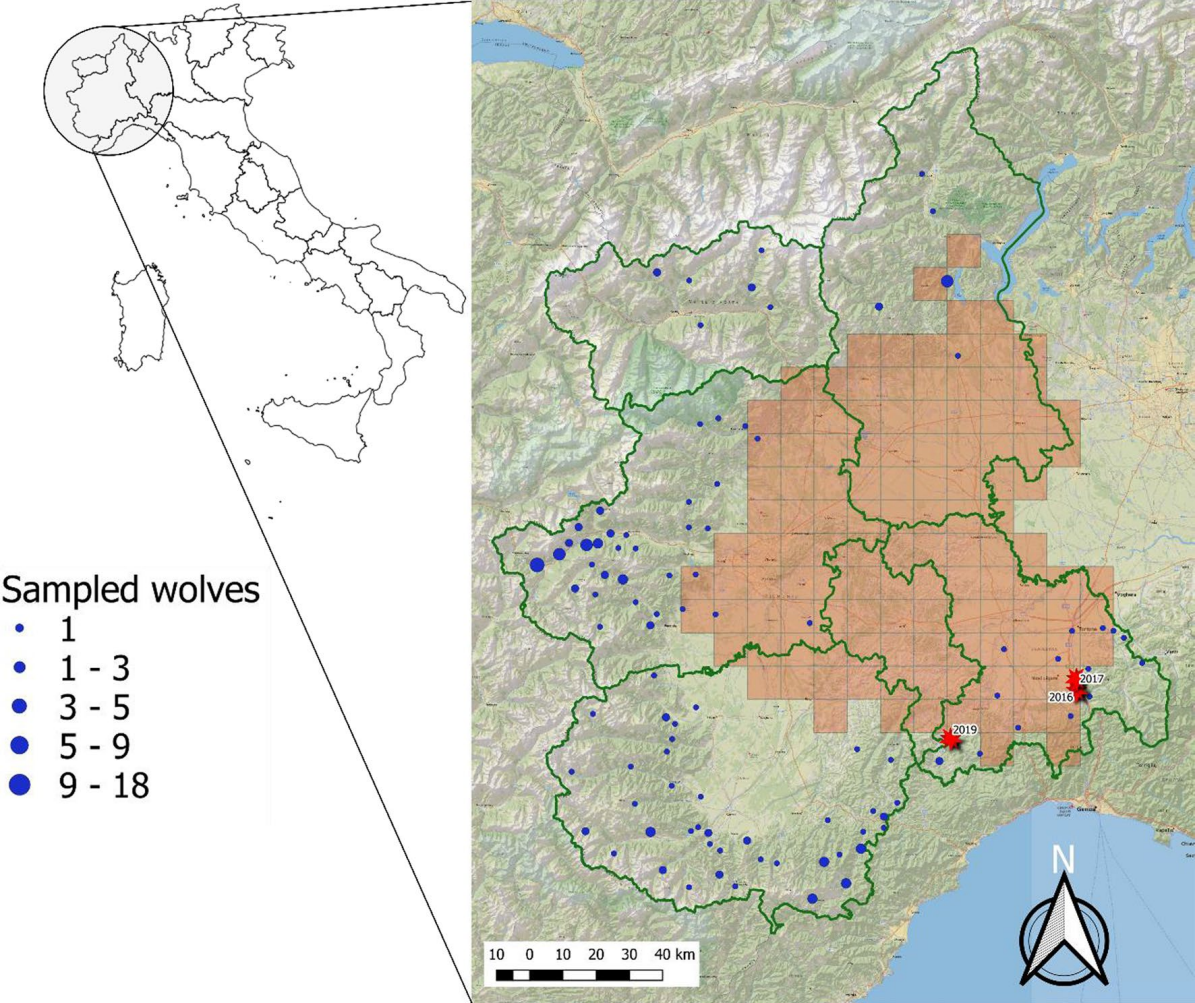
Origin of wolf carcasses collected in northwestern Italy. Blue dots represent the location of negative wolves. Pink represents the heartworm endemic area based on the study on unprotected dogs [2]. Stars indicate wolves infected with *Dirofilaria immitis*

**Fig. 2:**
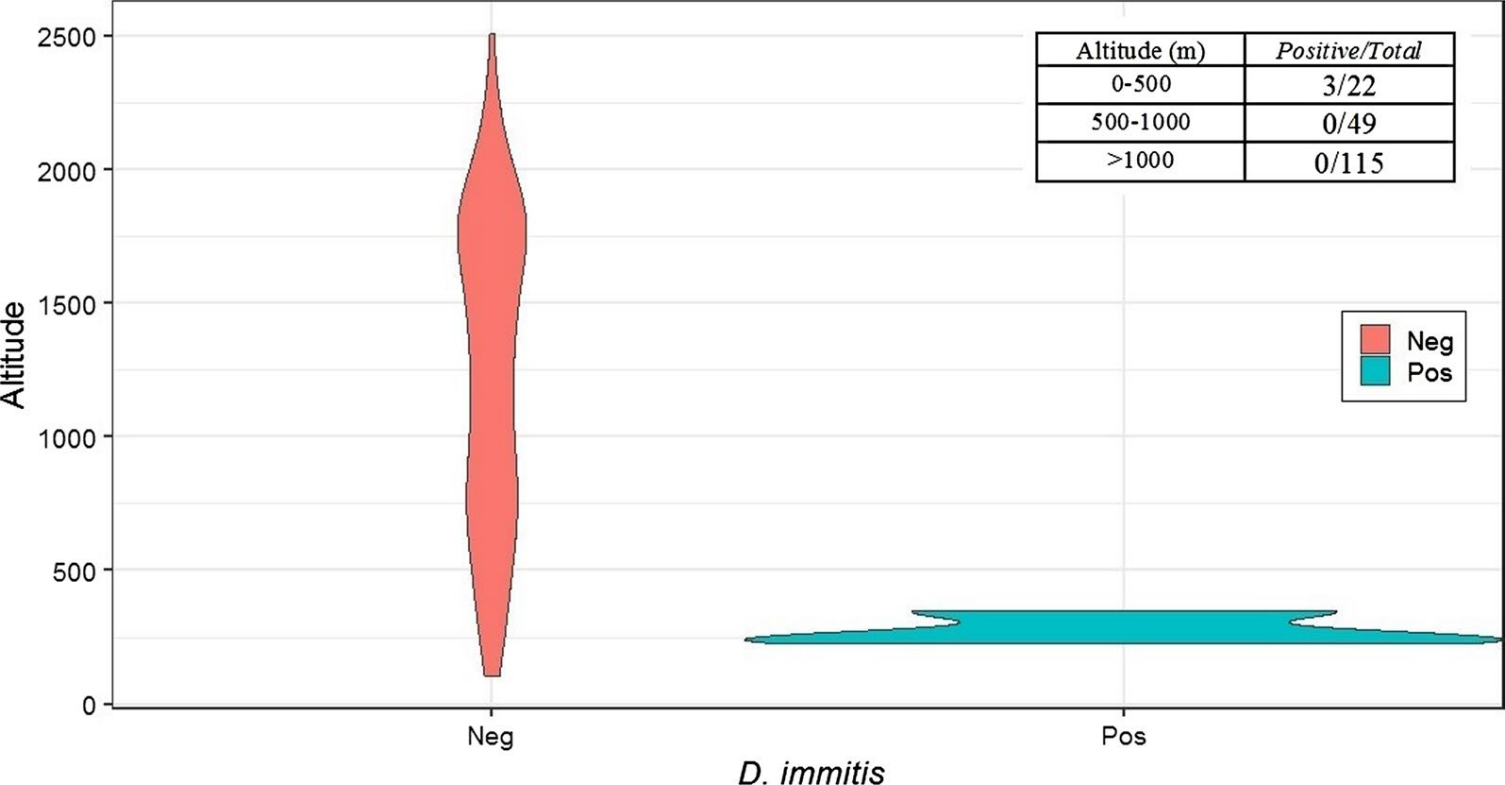
Violin plot illustrating the mean altitude of locations where heartworm-positive and negative wolves were collected

Infected wolves were in optimal nutritional status, as were the vast majority of uninfected wolves. They originated from a single province, Alessandria (*n* = 22; prevalence: 13.6%; 95% CI: 4.7–33.3%) (Fig. 1). The association between heartworm presence and the origin of wolves was significant (Mid-P exact test, *P* = 0.001012). Altitude was also significantly associated with the origin of infected wolves, with all the positive wolves (3 out of 22) reported in hill areas below 500 meters of altitude at the limit with low Apennine mountains (in the Alessandria province) (Wilcoxon rank sum test; *W* = 522,, *P* < 0.01) (Fig. 2). The violin plot shows the frequency distribution of positive and negative wolves in relation with altitude, highlighting the strict (and negative) relationship between altitude and presence of the parasite.

A study of unprotected dogs in the same examined area in northwestern Italy showed that heartworm prevalence in the Alps (where most of the examined wolves originated) was 0.2% whereas it was 27.3% and 7.8% in wolves sampled in hill zones and nearby Apennines, respectively [2].

No macroscopic cardiac or pulmonary arterial lesions were observed. One wolf had female worms only, whereas male and female nematodes were collected in the other two. The length of female heartworms ranged between 21–28 cm (mean ± SD = 24.4 ± 2 cm). Based on the embryogram (Table 2), 10 adult nematodes were ranked as follows: “non-gravid” (3 worms) in Wolf 3; “normal” (4 worms) in Wolf 2; mixed “normal” (1 worm) and “discontinuous gradient” (1 worm) in Wolf 1.

**Table 2:** Mean percentage of embryonic stages and embryogram classification of *Dirofilaria immitis* (HW) specimens recovered from 3 out of 210 wolves in northwest Italy

Positive wolf	Age class	Embryogram classification (HW females)	SM (%)	PR (%)	DE (%)	PL (%)
Wolf 1	Adult	Normal	14.6	20.4	26.2	38.8
		Discontinuous gradient	49.0	0	11.3	39.7
Wolf 2	Adult	Normal	8.7	28.8	28.1	34.4
		Normal	9.2	32.9	31.2	26.7
		Normal	34.4	28.0	30.6	7.0
		Normal	35.0	24.5	32.2	8.3
Wolf 3^a^	Subadult	Non-gravid	0	0	0	100
		Non-gravid	0	0	0	100
		Non-gravid	0	0	0	100

^a^No adult male HW were present in this wolf

*Abbreviations*: SM, stretched microfilaria; PR, pretzel; DE, developed embryo; PL, pre-larvae

**Fig. 3:**
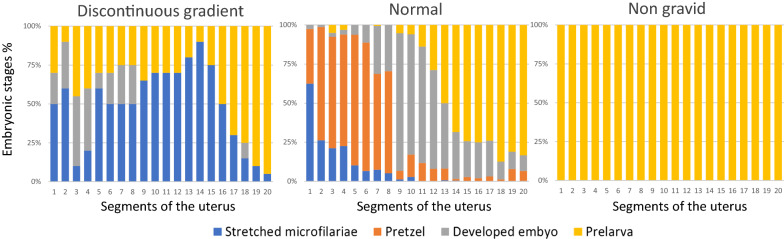
Example of three composite embryograms for investigated heartworm females. Each uterine segment is numbered from 1 to 20, from the head to the tail end. Right: non-gravid heartworm female, in which no stage beyond pre-larvae is present. Centre: “normal” gravid female in which all embryonic stages are present with a regular tail-to-head distribution from pre-larvae to stretched microfilariae. Left: “discontinuous gradient” gravid female in which microfilariae are present but other embryonic stages (e.g. pretzels) are missing

Microfilariae were found in two thirds of the examined adult nematodes. An example for each category of “embryogram” classification is provided in Fig. 3. Results show that gravidity is a common outcome among heartworm females hosted by wolves.

## Discussion

The main points of this study are: (i) the relatively high prevalence of *D. immitis* in wolves originating from the lowest altitude part of the recolonized range; and (ii) the evidence that wolves are suitable heartworm hosts.

Heartworm prevalence in the present study is low compared to similar studies (Table 3). In fact, the majority of examined wolves (*n* = 115, see Fig. 2) originated from the Alps, where temperatures are not or are rarely favorable to the completion of the *D. immitis* life-cycle in the potential mosquito vectors, although still compatible with the survival and hatching of mosquito eggs [35–37]. In mountain areas, activity of the potential vectors is also shorter than in lower altitude zones, often permitting no more than a single reproductive cycle per year [35]. In this study, the maximum altitude recorded for a heartworm-positive wolf was *c*.350 m above sea level, corresponding to hill zones at the limit with low mountains. In this regard, environment and climate influence on parasite distribution is commonly described [38, 39]. Further explanation for the low prevalence in investigated wolves is the age structure, with juveniles (< 12 months) summing up to one third of the sample (38.1%) (Table 1). Several studies of unprotected dogs and wild canids have identified adult age as a significant risk factor for heartworm infection [2, 40].

**Table 3:** An overview of heartworm studies in wolves (*Canis lupus*) worldwide

Country	Prevalence (%)	*n*	95% CI	Detection method	Year	References
Italy	1.4	210	0.5–4.1	N	2001–2019	Present study
	CR	1	–	N, MI	2003	[19]
Serbia	1.4	70	0.3–7.7	N	2009–2013	[7]
	CR	1	–	N	2014	[22]
Romania	0	14	–	MI	2014–2016	[13]
Spain	2.1	47	0.4–11.1	N	1993–1999	[17]
	0	74	–	N	2008–2014	[23]
Bulgaria	5.5	18	1–25.8	N	1997–1999	[18]
	0	3	–	N	2012–2013	[21]
USA (Minnesota)	6.2	387	4.2–9.0	N, S	2007–2013	[20]
USA (Wisconsin)	9.2	371	6.6–12.6	S	1985–2011	[10]

*Abbreviations*: N, necropsy; S, serology; MI, molecular identification; CR, case report

Interestingly, the prevalence of *D. immitis* in wolves originating from the province of Alessandria, the single origin of infected wolves in this study, was similar to the prevalence in unprotected sympatric dogs (13.6% of the present study compared to 7.8% reported in [2]). This finding suggests that, at lower altitudes, at the rural/sylvatic interface, wolves and dogs may be similarly infected by *D. immitis*. Previously, consistent results have been obtained for coyotes and sympatric dogs in the USA [11]. It is worth stressing that the prevalence of microfilaremic dogs in Rossi et al. [2] likely underestimated the actual heartworm prevalence, since the diagnostic method used could not identify dogs that, although parasitized by *D. immitis*, are amicrofilaremic hosts. In other *in vivo* studies in heartworm-endemic areas, in which antigen tests were used, amicrofilaremic dogs were shown to be positive in over 17% of cases [41].

Our study shows also that, in wolves with heartworm infections comprising both sexes, a high number of female nematodes reach sexual maturity and harbor uterine microfilariae (Table 2). This outcome, despite the small number of infected wolves, clearly shows the fertility of *D. immitis* in wolves, thus the competence of the wild host in the life cycle of the parasite. Similar results were obtained in studies of experimentally [31] and naturally infected dogs [3]. In addition, the size of adult nematodes from wolves was similar as reported in dogs (mean length: 25.5 cm) [3]. In contrast, studies on red foxes showed that the majority of female worms were small-sized immature individuals [5, 14]. A fox-like pattern was revealed recently in the golden jackal, a canid currently spreading from the Balkans into central Europe, including northern Italy [14]. Overall, the similarity with dogs and the substantial difference with other wild canids, suggest that wolves are fully competent hosts of *D. immitis* and in the future may represent a complementary reservoir of this parasite, aside unprotected dogs.

## Conclusions

The present data suggest that wolves are fully competent hosts of *D. immitis*, and show an infection prevalence similar to sympatric unprotected dogs. Nevertheless, further studies are necessary to infer the role of the wolf as a heartworm maintenance host, as shown for coyotes in the USA [8] and dingo (*Canis lupus dingo*) in Australia [42]. As clearly stated in [43], the critical issue when defining a candidate reservoir in a multi-host system is the persistence of infection in that particular host, which can only be determined through longitudinal studies. However, the results of the embryogram highlighted the successful reproductive capacity of *D. immitis* in wolves, and consequently, their potential role in the parasite epidemiology. In the future, it will be advisable to monitor heartworm infection in wolves in northern Italy, since: (i) global warming will likely favor the altitudinal spread of heartworm infection in the Alps [44, 45], which are the core area for the recovering wolf population in northern Italy [46]; and (ii) ongoing dispersal of wolves from the Alps to hill and lowland zones where heartworm is endemic in dogs [47, 48], will expose them to much stronger heartworm challenge. Under these circumstances, it is reasonable to foresee a greater impact of heartworm infection on the health, fitness and life-expectancy of wolves [46, 49]. Finally, wolves dwelling in heartworm endemic zones could raise the interest by practitioners and drug companies, since unprotected dogs are increasingly rare and not easy to detect. In this particular context, wildlife sentinels mirroring environmental exposure risk to heartworm infection would be welcome.

## Data Availability

The datasets used and analyzed during the present study are included in this article. Raw data used and analyzed during the current study are available from the first and corresponding author upon reasonable request.

## Abbreviations

CI: confidence interval
OR: odds ratio
SD: standard deviation
